# A Scoping Review of Homebound Older People: Definition, Measurement and Determinants

**DOI:** 10.3390/ijerph18083949

**Published:** 2021-04-09

**Authors:** Young Ko, Wonjung Noh

**Affiliations:** College of Nursing, Gachon University, Incheon 21936, Korea; moodory@gmail.com

**Keywords:** older adults, homebound, determinants, definition, scoping review

## Abstract

Being homebound (HB) can affect people’s physical and mental health by decreasing movement, which can itself be exacerbated by the deterioration of people’s health. To break this vicious cycle of HB and being in poor health, it is necessary to identify and address the factors influencing HB status. Thus, we used a scoping review to identify an HB trend, focusing on the definition, measurements, and determinants of HB status. We analyzed 47 studies according to the five-stage methodological framework for scoping reviews. The common attribute of definitions of HB status was that the boundaries of daily life are limited to the home. However, this varied according to duration and causes of becoming HB; thus, the understanding of HB shifted from the presence or absence of being HB to the continuum of daily activity. Various definitions and measurements have been used to date. Many studies have focused on individual factors to analyze the effect of HB. In the future, it will be necessary to develop a standardized measurement that reflects the multidimensional HB state. In addition, it is necessary to utilize a theoretical framework to explore the social and environmental factors affecting HB.

## 1. Introduction

The number and proportion of people aged 60 years or older is rapidly increasing worldwide. According to the World Health Organization, there were one billion people aged 60 or older in 2019. The number of older people is expected to increase to 1.4 billion by 2030 and 2.1 billion by 2050 [1]. This increase in the older population can also lead to an increase in the proportion of homebound (HB) people who stay at home [2].

HB people suffer from multiple physical health problems, such as metabolic, cardiovascular, cerebrovascular, and musculoskeletal diseases, as well as psychiatric health problems, such as cognitive impairment, dementia, and depression [3]. Despite the high demand for medical care services, their access to healthcare services is limited because of their physical and economic dependency coupled with a lack of information and support [4]. In addition, because they are noncompliant with medication adherence and care pattern rules, they use healthcare services more and spend more on health management than those who are not HB [5]. Existing social networks may be reduced or eliminated because they are HB [6]. Loneliness may arise when there is a mismatch between desired and available forms of social and emotional connections [7]. Therefore, HB status is a possible risk factor that increases social isolation and loneliness. Eventually, HB leads to negative health outcomes such as mortality [8,9] and a low quality of life [10]. HB adversely affects health [8], and when health deteriorates, it causes HB again [11]. To avoid this vicious cycle of HB and poor health, it is necessary to explore the causes and status of HB.

Several countries have services to manage HB. In the United States, skilled nursing care, physical therapy, speech-language pathology services, and continued occupational services have been provided to HB people [12]. Singapore also provides home-based services such as medical care, nursing care, personal care, therapy, palliative care, meals on wheels, and transport/escort services to the HB elderly [13]. In European countries, a proportion of older adults, including frail and ill older adults, receive home care [14]. For example, Sweden provides primary health care by a physician and visiting nurse and home help, including social care and personal care, to older adults staying in their own homes [15].

However, these services have only focused on solving problems caused by HB, and there is a lack of preventative services. In Japan, the focus was initially on troubleshooting HB, but it was changed to focus on prevention because continuous service, which focused on problem solving, was recognized as limited [16]. Therefore, it is important to understand the current situation, as well as interventions for older adults with HB and prevention of HB among older adults.

Studies on the prevalence of HB and its influencing factors have been conducted since the 1990s [17,18]. In an early study, HB was defined as being confined to the home [17]. In previous studies, it was sometimes defined as not having gone out for a certain period of time, such as a week or month [19,20]. In addition, the definition of HB is not only related to the concept of time, but also to the cause of HB, such as the absence of assistance from others to go out or impairments of function to go out [18,21]. The definition of HB has changed over time. Thus, there are limitations in comparing the results between countries and studies. For example, in the United States, the HB prevalence rate among non-institutionalized Medicare beneficiaries aged 65 years and older using one question about the frequency of going out (completely or mostly HB) was 5.6% [22]. Another U.S. study defined HB as self-reported ambulatory disability without any restriction of additional medical needs; 19.6% of older adults were HB [5]. In Japan, the prevalence rate of HB in 5000 older adults, randomly extracted using one item with frequency of going out being less than once a week, was 14.4% [23]. In France, the prevalence of HB was 4.7%, measured by asking persons if they are usually compelled to stay inside the home permanently (excluding an accident or temporary illness), but are not bedridden [9]. HB was divided into HB and non-HB and then further subdivided into HB, semi-HB, and non-HB [20,22,24,25,26]. As the classification criteria for semi-HB has varied in previous studies, the prevalence of semi-HB also varies [20,22,24,25,26]. Thus, an in-depth review of the definition and measurement of HB is needed prior to conducting studies to develop interventions to prevent HB.

Previous studies have investigated factors affecting HB in various ways, such as personal [19,23,27,28,29,30], social [2,31], and environmental factors [32]. Along with the changes in the definition of HB, it is expected that the view of HB’s influencing factors will also have changed; however, few studies have reviewed the factors affecting HB in older adults. HB can be influenced not only by individual factors but also by social relationships, social support systems, and environmental factors [33]. Therefore, it is necessary to review the factors affecting HB using an ecological approach [20]. Through this, we propose the implications of and an approach to interventions to prevent HB.

Scoping review is a new methodology for synthesizing the results of previous studies. It is excellent at identifying differences in knowledge, broadening knowledge, clarifying concepts, and investigating results [34]. It can provide an overview of a concept, and is useful in answering broad research questions [35]. Therefore, in this study, it was considered appropriate to conduct a scoping review to explore how the definition of HB has changed, how it is being measured, and the related factors that influence it.

## 2. Materials and Methods

### 2.1. Design

This scoping review utilized the five-stage methodological framework for scoping studies: (1) identifying the research question, (2) identifying relevant studies, (3) selecting studies, (4) charting the data, and (5) summarizing and reporting the findings [36]. We performed the study in accordance with these stages and performed Steps 4 and 5 simultaneously.

### 2.2. Identifying the Research Question

This review aimed to answer three questions: (1) How is HB defined? This question aimed at determining how definitions differ by various components, such as the times or regions. (2) How is HB measured? Through this question, we tried to identify any differences in the measurement and methods of HB. (3) What are the factors that influence HB? The last question was aimed at identifying the factors affecting HB, and to classify them systematically. We did not define HB because we wanted to compare the definitions and measurements used in previous studies. In addition, to answer our research questions, we included a broad range of determinants and categorized them based on the revised ecological model [20].

### 2.3. Identifying Relevant Studies

We searched four databases, including a domestic (Korean) database and three international databases. The domestic database used was the Research Information Sharing Service, and international databases were PubMed, the Cumulative Index to Nursing and Allied Health Literature database, and Embase. A literature search was conducted for two weeks from 30 January 2020. We used a combination of search terms referred to in the PICO model. Regarding inclusion criteria, older people were selected for the study. Interventions and comparisons were not considered in this study. As our aim was to explore the definition, measurement, and factors affecting HB, we did not establish an intervention and comparison group to explore studies using various research. Finally, in order to explore the influencing factors of HB, a study with HB as the outcome was considered in the inclusion criteria. The following search terms were used based on the MeSH (Medical Subject Headings) term: “aged” AND (“social isolation” OR “homebound person”) AND “health” The reason the keyword was set to “aged” without limiting the target age to above 65 was because some countries defined those aged 50 or older as old adults. Data retrieval was conducted without limiting the publication date.

### 2.4. Selecting Studies

We selected the papers according to the following inclusion criteria: language, design, publication type, and outcome. We included both English and Korean studies and all research designs. We only included published articles, excluding editorials, book reviews, and poster presentations. In addition, we included a wide range of studies related to older people’s HB. We selected papers for two-step screening by title, abstract, and full text.

**Figure 1:**
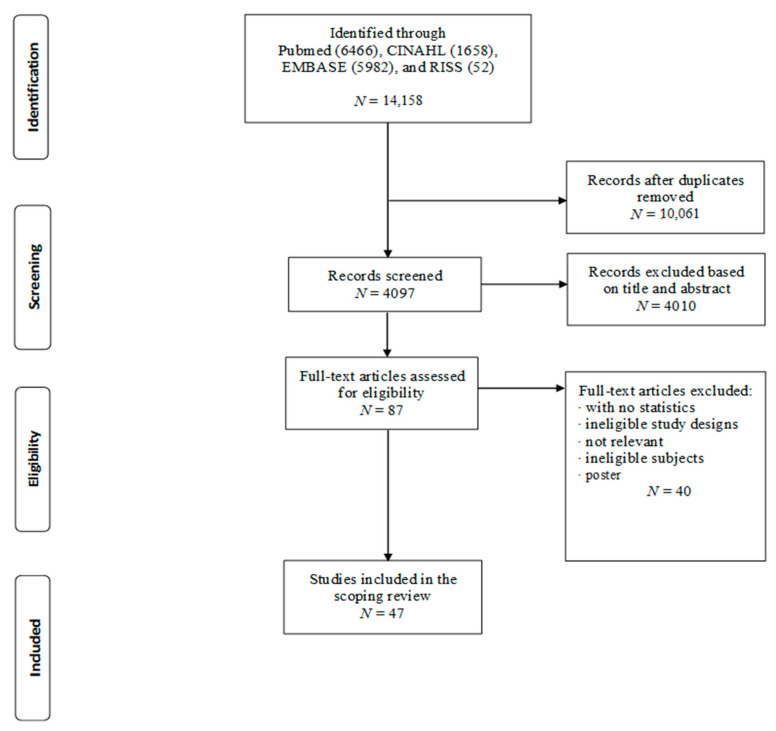
PRISMA flowchart of article selection.

The selection process is illustrated in Figure 1. A total of 14,158 articles were identified from the four databases. Of the total number of articles, 10,061 duplicate articles were removed. After screening the titles and abstracts, 4010 articles were excluded, leaving 87 articles. After checking for eligibility, 47 articles were finally included in the scoping review.

### 2.5. Charting the Data and Summarizing and Reporting the Findings

Each eligible study was charted and summarized using a standardized form, including author, publication year, design, participants, age, number of participants, country, definition, and theoretical model. In addition, the definitions of HB and the measurements used were analyzed. The measurements of HB were charted according to the measurement period, frequency, and meaning of “outdoor activities.” The factors affecting HB were analyzed according to the framework presented in Table 1. The framework consisted of authors referring to the revised ecological model [20] and three factors: individual, social, and environmental. There were six categories of individual factors: demographic, health characteristics (illness), physical function, psychological function, cognitive function, and health behavior.

## 3. Results

### 3.1. General Characteristics of the Study

The general characteristics of previous studies regarding HB are shown in Table 2. Two-thirds of the 47 studies were cross-sectional studies, and 68.1% were older people. Regarding age, 78.7% of the participants were over 65 years, and the number of participants was below 1000 in 46.8% of the studies. Most of the studies were conducted in the United States and Asia, and 34.0% have been conducted since 2010. Among the studies, 72.3% presented a definition of HB, and 8.5% used a theoretical model.

### 3.2. Definition of HB and the Applied Conceptual Model

Table 3 shows studies that present the definition of HB and the applied conceptual framework of HB. Most researchers defined HB as “a condition characterized by an infrequency of going outdoors” [6,9,19,20,21,22,26,32,37,38,39]. Other researchers defined HB as being “confined to one’s home” [17,18], “remaining inside or just around the home [during] daily life” [40], or “being compelled to stay inside one’s home permanently excluding an accident or temporary illness” [9].

In some studies, the cause or situation of HB was specified in the definition of HB [18]. The boundary of the daily life of older adults is limited to home because of immobility [18,21,40], medical issues [21], or either help or taxing effort is needed to leave the house [21,40]. Koyama and colleagues defined the reason for being HB as not only physical but also psychological or geographical [6]. Chinese researchers [39] viewed HB as a continuous spectrum (line) of living activities. They included, in the concept of HB, not only being physically confined to the house, but also the degree of help needed to leave the house and whether an individual had social contact.

The application of a conceptual framework to explain HB began in 2001. HB is closely related to the aging process [40]; thus, HB refers to a condition in which physical and social independence are impaired, and dependence on others increases due to chronic physical illness and disability. Ornstein et al. applied a gerontological conceptual framework to explain HB. They explained that the impact of disability is based on the confluence of personal capacity and the ability of social support to compensate for capacity limitations [22]. Xiang et al. explained HB using the International Classification of Functioning, Disability, and Health (ICF) and the concept of autonomy [32]. They defined HB as a result of interactions between body functions and structures, activities, participation, and environmental factors [6,41]. In other words, HB is defined as one of the states of a continuum of living activities. Xiang et al. applied a revised ecological model of aging [32] to determine the influencing factors that cause HB [20]. These include demographic (age, gender, race, and ethnicity), socioeconomic (such as education and income), social (such as living arrangements, social networks, and social support), and psychosocial factors (e.g., self-efficacy), health behaviors, health conditions, functioning (physical and cognitive), and environment (built and physical). This is a point of view that considers not only individual factors but also social and environmental factors as the reasons for HB.

### 3.3. Operational Definition of HB

Most epidemiological researchers have investigated HB using self-reported questions about the frequency of going out for a certain period. The observation period for measuring the frequency of going out varies from one week [28,37,50] to one month [18,20,26,27,30] or usually [9,47]. The criteria for HB also varies according to the study, from not going out at all in some studies [9,18,28,40,50] to going out less than once [2,8,10,19,20,22,23,29,37,38,44,47]. When defining HB, the scope of the house also varies, from staying inside the building or staying on the block where the house is located (blockbound) [18] to staying in the house, including the garden or yard [38]. The meaning of “going out” varies as well. The activity of leaving the house to take out the trash is not considered going out, but activities that involve leaving home for a certain purpose, that is, going shopping, walking for leisure, visiting the hospital or center, commuting, and going to work or participating in social activities, were defined as going out [28,39]. In some studies, emergency visits due to accidents or temporary illnesses were not included in “going out” [9].

Researchers [20,22,26] in the United States have classified HB into three groups: HB, semi-HB, and non-HB. HB was defined as never or rarely leaving home. Semi-HB refers to cases where individuals get or need help to go out, or it is difficult for them to go out by themselves. Non-HB is a case in which a person can go out without help. Meng et al. defined HB as going out less than once per day and calculated the severity of HB by adding up the amount of time a person spent at home; the frequency at which they went out, met, and communicated with friends, neighbors, and relatives outside their home; and how often they needed help when going out [39].

### 3.4. Factors Affecting HB

For the analysis of determinants, we categorized three factors based on the revised ecological model: individual, social, and environmental factors (Table 4). The individual factors were divided into six categories: demographic, health characteristics, physical function, psychological function, cognitive function, and health behavior. The effects of personal factors were investigated in all studies. Of the 16 studies, 10 (62.5%) were identified as exploring the influence of social factors, and 6 (37.5%) as exploring the influence of environmental factors.

**Figure 2:**
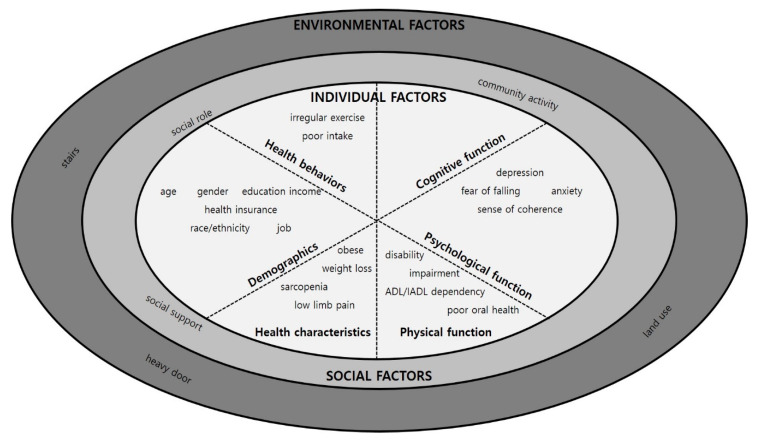
Significant determinants of HB.

The statistically significant determinants of HB are shown in Figure 2. For the individual factors, there were significant variables in the demographic categories: age, gender, education, income, health insurance, race/ethnicity, and job. Among health functions, there were significant variables: sensory impairment, mobility impairment, disability, and activities of daily living/instrumental activities of daily living (ADL/IADL) dependency. In addition, irregular exercise and poor intake were significant in the health behavior category. In the physical category, weight loss, obesity, sarcopenia, oral health, and low limb pain were significant. In addition, there were significant variables: depression, fear of falling, anxiety, loneliness, and sense of coherence. Social support, social roles, and the community were significant social factors. For environmental factors, stairs, heavy doors, and land use were significant variables.

## 4. Discussion

This study identified the definition, measurement, and determinants of HB through a scoping review. We discuss these points and suggest their implications.

First, as a result of the scoping review, we found two definitions of HB to be widely used in the studies: “confined to home or remain inside the home” and “infrequently going outside the house.” The former, home confinement or remaining inside the home, occurs due to a decline in an individual’s ability to move, a medical problem that makes it difficult to move, and/or a lack of resources to help an individual who cannot move [17,21]. This definition was used in early studies [9,17,18,41] and in studies defining targets for providing long-term care services in Japan [52] or Medicaid in the United States [17,21]. This definition is in line with the gerontological conceptual framework that the impact of late-life disability is based on the confluence of personal capacity and the ability of social support to compensate for limitations in capacity [53,54]. In contrast, “infrequently going outside the house” is presented as the result that reflects the individual’s will and surrounding environment as well as their ability to move [47]. This definition is widely used in epidemiological studies [23], especially in Japan [2,29,38,49].

Through two HB definition analyses, we found that the common attribute of HB in previous studies is “the boundary of daily life is limited to home.” Life–space mobility encompasses a person’s independent mobility, requiring mobility-related physical activity (e.g., walking), and all movements supported by mobility aids and/or means of transportation [55]. Life–space mobility limited to home is similar to the common attributes of HB. Increasing living spaces would promote more activities and increase people’s well-being [56]. Therefore, it is important to understand the meaning and boundaries of older adults’ daily lives to prevent HB.

Second, to measure or assess HB, a question about the frequency of going out was used in most epidemiological studies. However, the observation period for measurement, the definition of “going out,” and the criteria for determining HB were different. For this reason, the prevalence of HB varies from 3.5% [18] to 26.5% [37]. Gilber et al. examined the validity of one question, but the cut-off was “never or almost never except for emergencies on a six-point scale of frequency (almost every day, a few times a week, once a week, several times a month, less than several times a month but more than just for emergencies, and never or almost never except for emergencies)” [12]. Although one study reported that rarely going out has a higher probability of mobility disability [57] and death [58] than going out daily, previous researchers used a variety of cutoffs for the frequency of going out, which is the criterion of HB [9,39,50].

Some researchers have classified HB, beginning with the idea that HB people may have different health effects depending on the characteristics, prognosis, and causes of HB. For example, some researchers categorized HB into three groups based on the availability of help for going out and the difficulty of going out [26]. Another researcher [38] considered social isolation to be an important aspect of HB and classified HB into four groups: immobility and social exchange. However, some researchers have recently viewed HB as a continuous and dynamic process. For example, Xiang et al. defined HB as a continuum of outdoor mobility determined by physical capacity, availability of social support, and degree of autonomy [32]. Meng et al. evaluated the severity of HB based on the frequency of social contact, frequency of going out, time spent at home, and the need for help to go out [39]. When HB status is assessed, it is very important to consider the meaning of HB and to recognize HB as a preventable and manageable health problem. In addition, rather than simply screening HB people who stay at home all the time, it is necessary to develop a multidimensional tool that screens high-risk groups and provides appropriate interventions to prevent HB. For HB status assessment and comparison between nations, it is necessary to be able to measure and compare HB prevalence rates using standardized measurements.

Finally, we regard HB as a health outcome and identify the influencing factors of HB in older adults based on the revised ecological model. The theoretical framework for explaining HB was also applied in line with the changes in the definition of HB and its determinants. In a previous study, a conceptual framework related to the aging or disability process was applied to explain HB [26]. However, recent researchers have tried to explain HB and its influencing factors by applying an ecological model [20] or the ICF model [32]. Although few studies have applied the conceptual framework to preceding factors and the consequences of HB, it is interesting to focus on HB due to social issues or environmental factors.

As a result of classifying related variables using the revised ecological model, most researchers focused on individual factors, including health characteristics, physical function, and psychological function. Early researchers considered individual factors such as immobility or medical conditions, psychological issues, and decreased autonomy as the causes of HB. In addition, physical function and physical illness should be managed to prevent HB in older adults because HB occurs due to physical limitations or lack of social support for going out. However, recent studies have reported that HB occurs due to not only individual factors, but also social factors [2,18,19,23,27,29,30,31,32,44] or environmental and geographic characteristics [6,18,29,32,47,50]. Therefore, it is necessary to identify the various factors that influence HB by applying a theoretical framework.

Our findings have important implications for the expansion of the current view of HB research. We suggest that determinant survey is based on a theoretical model, especially from social and environmental perspectives. Individual factors have already been studied, but social and environmental factors have not been studied. The effects of various social factors such as social role, community participation, social networks, social isolation, and social capital on HB need to be identified.

As a result of this scoping review, social role, social support, and community participation among social factors have been identified as factors influencing HB status. In a previous study, HB increased loneliness and social isolation, which has been reported to worsen mental health among HB older adults [20,59]. Given that both social isolation and HB are strongly related to an increased risk of mortality, the coexistence of social isolation and HB may increase the risk of mortality as a synergistic effect [6]. Therefore, to prevent or manage HB, it is necessary to recognize the importance of establishing a support system to maintain social relationships and participation and develop formal social support services to help people go out regularly.

In South Korea, long-term care recipients mainly use housekeeping and living support services at home among the care services offered by the Korea Long-Term Care Insurance system [60]. In contrast, since April 2006 in Japan, people with HB have been specified as eligible for public long-term care insurance (LTCI), and LTCI has provided nursing and care services for HB people, as well as HB prevention services [16]. Therefore, there is a need to develop a service to prevent HB in older adults and provide proper home-based primary services for HB adults in long-term care services.

Recent studies have considered environmental factors, including the physical environment in the house, the residential area, the availability of transportation facilities, and the perception of neighborhood environment as influencing factors of HB. The scoping review showed that, among the environmental factors, neighborhood environment and physical obstacles to leaving the house, such as heavy doors and the presence of stairs, were significant factors influencing HB [29,50]. Therefore, to prevent HB, it is necessary to support environmental improvement projects in homes where older adults reside. In addition, communities with extensive transportation networks and walking access to various non-residential properties are ideal for encouraging the elderly to go outside their homes.

This study has several limitations. First, we set the inclusion criteria to focus on the general HB. HB-related disabilities and diseases, such as mental health problems, were excluded from the study. This was done to focus on older people’s HB, but in order to identify HB as a whole, it is necessary to expand the inclusion criteria in further research. Second, the perspective regarding HB has changed; so, it is important to recognize that this review represents a slice of information at one point in time. Third, we included only English-language abstracts. Papers initially written in Japanese were not included in this review. In addition, we did not include gray literature or doctoral theses. Finally, we conducted a scoping review. Therefore, we did not evaluate the quality of paper, publication type, etc., and we excluded papers based on their quality.

## 5. Conclusions

This study was conducted to identify the definition and determinants of HB and its recent trends through a scoping review. The definition and classification of HB is constantly changing and expanding as a result of changing perspectives on the determinants of HB. Initially, HB was defined to clarify the specific service target, but its definition and classification gradually changed as HB came to be regarded as a health problem that needs to be prevented and managed. Based on the results of this study, we provide the following suggestions and implications. First, it is necessary to develop a multidimensional tool to measure HB so that it cannot only screen individuals at risk of becoming HB, but also suggest interventions for preventing and managing HB. Prior to this, an in-depth study should be conducted on the meaning and pattern of the boundary of daily life for older people in a cultural context. Second, the factors influencing HB, particularly individual factors, have been studied. The importance of environmental and social factors is emerging as an important determinant of HB. Therefore, it is necessary to identify the various factors that affect HB by applying a theoretical framework. Finally, as society ages, an increase in the number of older people who need long-term care, including HB individuals, has emerged as a major social problem. Living in “the boundary of daily life is limited to home” will negatively affect older adults’ health and decrease their quality of life, while incurring enormous social costs to provide older adults with adequate services. Therefore, there is an urgent need to develop health policies to prevent HB in older adults.

## Figures and Tables

**Table 1:** Analysis framework of determinants of HB.

Factor	Category	Examples
Individual factor	Demographic	Age, gender, race, marital status, education, occupation, income, health insurance, etc.
	Health characteristics	Height, weight, body mass index, comorbidity, illness, sarcopenia, laboratory analysis, etc.
	Physical function	Activities of daily living, Instrumental activities of daily living, mobility difficulty, hearing and vision ability, etc.
	Psychological function	Mental health, depression, anxiety, phobia, etc.
	Cognitive function	Cognitive impairment
	Health behavior	Exercise, eating habits, smoking, drinking, etc.
Social factor		Social participation, social support, social activity, social capital, social isolation, contact with various community health and social services, etc.
Environmental factor		Use of a mobility device to get around, stairs or steps at the entrance, population size, size of area, etc.

**Table 2:** General characteristics of previous studies regarding HB (*N* = 47).

Characteristics	Category	*N*	%
Design	Longitudinal study	16	34.0
	Cross-sectional study	31	66.0
Participants	Older people	32	68.1
	HB older people	15	31.9
Age	Above 50–64	10	21.3
	Above 65	37	78.7
Number of participants	≤1000	22	46.8
	1000~2000	8	17.0
	≥2000	17	36.2
Country	Asia	18	38.3
	Europe	9	19.1
	America	20	42.6
Publication year	2010-present	16	34.0
	2001–2009	31	66.0
Definition	Yes	34	72.3
Theoretical model	Yes	4	8.5

**Table 3:** Definition of HB and the applied theoretical model.

Author	Year	Country	Definition (Conceptual Framework)	Operational Definition	Going Out of the House		
					Observation Period for Measure	Frequency	Meaning of “Outdoor Activities”
Bruce & McNamara [17]	1992	US	Being confined to the home	Two items: individual stayed in their bed or in a chair for most or all of the day during the last two weeks or they stayed indoors for most or all of the day during the last two weeks.	2 weeks		
Lindesay & Thomson [18]	1993	UK	Being neither completely housebound nor blockbound or only going out of doors with the assistance of others	Individuals were considered housebound if they met the following two criteria: (1) they were either completely housebound or blockbound (for at least one month) and (2) interviewers judged that their current housebound or blockbound status was likely to be permanent.	1 month	Never	Individual goes beyond their door or the boundary of the block
Ganduli et al. [19]	1996	US	Leaving the house once per week or less	One item: “How often do you get out of the house?”		Once per week or less	
Engberg et al. [21]	2001	US	Being confined to the home Homebound (HB)older adults are those who, due to medical conditions and/or mobility-affecting impairments, are not able to freely leave their homes, and require help in doing so	Health Care Financing Agency Criteria			
Kono & Kanagawa [37]	2001	Japan	Being HB was defined according to the frequency of getting out and mobility.	Self-reported behaviors (10 behaviors) regarding getting out within a week	1 week	Never/once per week	Ten behaviors: go to adult day care, going out to the garden, taking a walk, and so on.
Inoue et al. [40]	2001	Japan	The state of remaining inside or just around the home during daily life (Aging process: the development of a chronic physical illness and disability in the elderly often results in a loss of physical and social independence and increased dependence on others)	One item: “In your daily life, do you leave home without assistance from others?”		Never	
Sharkey et al. [42]	2002	US	Medicare classified a person as HB if leaving the home requires considerable effort and occurs infrequently due to an illness or injury.				
Kawamura et al. [37]	2005	Japan	A condition characterized by an infrequency of going outdoors Four categories of HB: by social contract with friends, neighbors, or relatives other than live-in family members and with/without assistance	One item: frequency of leaving the house (1) once per day or more, (2) once every 2–3 days, (3) about once per week, (4) rarely.		once per week or rarely	Leaving the house including going to the surrounding gardens or grounds
Choi & McDougal [43]	2007	US	HB older adults are those who, due to medical conditions and/or mobility-affecting impairments, are not able to freely leave their homes and require help in doing so				
Katsumata et al. [44]	2007	Japan		One item: frequency of going outdoors		Once per week or less	
Locher et al. [45]	2008	US	Medicare definition of HB status: [An] individual [who] has a condition … that restricts [their] ability to leave home except with the assistance of another individual or the aid of a supportive device or [who] has a condition [where] leaving home is medically contraindicated				
Cohen-Mansfield [8]	2010	Israel		One item: how often do they go outside of their home (more than once per week, or once a week or less)		Once per week or less	
Murayama et al. [29]	2012	Japan		One item: “How often do you usually go outside the house?” (once a week or less vs. more than once per week)	Usually	Once per week or less	
Cohen-Mansfield [46]	2012	Israel		One item: “How often do you go outside of your home?” (more than once per week, or once a week or less)		Once per week or less	
Choi et al. [28]	2012	South Korea		One item: “How often do you go outside of your home?” (more than once per week, or once a week or less)	1 week	Never within a week	Go shopping or walking, visit the hospital or center (excludes leaving the house for a short period, for instance, to take out the trash)
Herr et al. [9]	2013	France	Remained inside their homes during the previous week or if they went out at all, only for health care purposes	One item for the non-bedridden: “Are you usually compelled to stay inside your home permanently (excluding an accident or temporary illness)?”	Usually	Never within a week	Accident or temporary illness
Umegaki et al. [23]	2015	Japan		One item: frequency of excursions within a week		Less than once per week	
Musich et al. [5]	2015	US	Ambulatory disability without the restriction of additional medical needs	Self-reported ambulatory disability (5 items): HB state was identified by answering “yes” to any of the following five items: (1) have trouble getting around at home or outside your home; (2) use a cane, wheelchair or walker to move around at home or outside your home; (3) need help from another person to move around inside or outside your home; (4) need to stay in the house most or all of the time; (5) need to stay in bed most or all of the time		Any ambulatory disability	
Ornstein et al. [22]	2015	US	HB: never or rarely left home; semi-HB: only left home with assistance or had difficulty or needed helping to leave home (Gerontological conceptual framework: late-life disability – the impact of disability is based on the confluence of personal capacity and the ability of social support to compensate for capacity limitations)	Three items: (1) how often they left home to go outside in the last month (daily, most days (5–6 days per week), some days (2–4 days per week), rarely (once per week or less), or never); (2) they were asked whether they needed assistance; (3) they were asked if they were ever able to go out by themselves, or they reported going outside without help then reported whether they had difficulty doing the activity alone (regardless of the use of assistive devices) in the last month.	Previous month	Never or rarely (≤1 day) within a week	
Takhashi et al. [31]	2015	Japan	A condition characterized by an infrequency of going outdoors	One item: frequency of going outdoors		Once per week or less	
Koyama et al. [47]	2016	Japan	Leaving home less often than once weekly, which reflects not only physical reasons for being confined to one’s home, but also psychological or geographical reasons	One item pertaining to frequency of going outdoors: “How often do you usually go outside the house?”	Usually	Less than once per week	Shopping, meeting up with people, walking, visiting the hospital, and other activities
Negron-Blanco et al. [30]	2016	Spain	Having severe or extreme difficulty getting out of the house	One item from WHODAS-36: “In the past 30 days, how much difficulty did you have leaving home?” None/mild/moderate/severe/extreme or cannot do, with a response of “severe” or “extreme or cannot do” was construed as being HB.	Past 30 days	Severe or extreme/cannot leave home	
Hamazake et al. [10]	2016	Japan		One item: “Do you go out more than once per week?”		Less than once per week	
Harada et al. [48]	2016	Japan	Going outdoors less than once per week	One item: “Do you usually go outside the house at least once per week?”	Usually	Once a week	
De-Rosende [49]	2017	Spain	Considered HB state if the individual remained inside their home during the previous week or if they went out only for health care purposes (e.g., medical consultation or health emergencies)	One item: the number of days on which they left home during the previous week	Previous week	Never or only for health care purposes	
Soones et al. [26]	2017	US	HB (never or rarely left home in the last month), semi-HB (only left home with assistance; needed help or had difficulty), non-HB (left home without help or difficulty) (Aday and Andersen’s Behavioral Model of Health Service Use and gerontological frameworks for the study of HB state due to disability)	Three items: “How often did you go out in the last month?” “Did anyone ever help you?” and “How much difficulty did you have leaving the house by yourself?”	Previous month	Never or rarely	
Jing et al. [27]	2017	China	People who leave home less than once per week	Record of occasions on which the individual went out during the month before the survey (In cases where the going out counts for each week of the month differed, the total count for that month averaged by the number of weeks was considered)	Previous month	Less than once per week	
Xiang & Brooks [25]	2017	US	“Never” or “rarely” left home in the last month HB, semi-HB: 1) they received help leaving home and would “never,” “rarely,” or “sometimes” go outside by themselves or 2) they did not receive help leaving home but reported “a lot,” “some,” or “a little” difficulty leaving home by themselves)	Three items: (1) how often they left home to go outside in the last month [daily, most days (5–6 days per week), some days (2–4 days per week), rarely (once per week or less), or never]; (2) they were asked whether they needed assistance; (3) they were asked if they were ever able to go out by themselves, or they reported going outside without help then reported whether they had difficulty doing the activity alone (regardless of the use of assistive devices) in the last month.	Previous month	Never or rarely (≤1 day) within a week	
Uemura et al. [2]	2018	Japan	Going outdoors less than once per week	One item: “Do you go out at least once per week?”		At least once per week	
Meng et al. [39]	2018	China	Going out of the house once per week or less	Four items: (1) “Do you spend more time at home than going out, and is this the norm?” (2) “How many times do you go out to shop, walk, or visit the hospital?” [(1) More than once per day, (2) 2–3 days at a time, (3) once per week, (4) hardly ever go out] (3) “How often do you meet or communicate with friends, neighbors, or relatives outside the home?” [(1) 2–3 days at a time (2) once per week (3) once per month (4) hardly ever], (4) “If you go out, do you need help?” Higher scores indicate severity of the HB status.			
Sakurai et al. [6]	2019	Japan		One item: “How often do you usually go outdoors?” (Twice daily or more, daily, about once every 2–3 days, about once per week or less often)	Usually	Every few days or less within a week	Going shopping, talking a walk, visiting the hospital, or going out to work or to participate in social activities
Zhao et al. [24]	2019		Participants were defined as totally HB if they never or rarely left home. Semi-HB participants were those who needed help leaving home and would “never,” “rarely,” or “sometimes” go out by themselves, or they had “a lot,” “some,” or “a little” difficulty going out by themselves without help.	Three items: (1) how often they left home to go outside in the last month [daily, most days (5–6 days per week), some days (2–4 days per week), rarely (once a week or less), or never]; (2) whether they needed assistance; (3) if they were ever able to go out by themselves, or they reported going outside without help then reported whether they had difficulty doing the activity alone (regardless of the use of assistive devices) in the last month.	Previous month	Never or rarely (≤1 day) within a week	
Xiang et al. [32]	2020	US	Never or rarely leave the house. HB status defined on a continuum of outdoor mobility determined by physical capacity, availability of social support, and degree of autonomy. Determined the classification of HB state (HB, semi-HB, or non-HBd) by the frequency of going out, physical capacity, availability of social support, and degree of autonomy. (International Classification of Functioning, Disability and Health and the concept of autonomy)	Four items: (1) “How often did you go out in the last month?” (responses on a 5-point Likert scale: never, rarely, some days, most days, every day) (2) “Did you ever have to stay in because no one was there to help you?” (3) “Did anyone ever help you?” (4) “How often did you go outside by yourself?”	Previous month	Never or rarely (≤1 day) within a week	
Xiang et al. [20]	2020	US	Never or rarely went out of the home in the last month (An ecological model revised by Satariano [50])	One item: “How often do you go outside of your home?”	Previous month	Never or rarely (≤1 day)	

**Table 4:** Determinants of HB state based on individual, social, and environmental factors.

Author	Year	Individual						Social	Environmental	Framework
		Demographic	Health Characteristic	Physical Function	Psychological Function	Cognitive Function	Health Behavior			
Lindesay et al. [18].	1993	O	O	O	O			O	O	
Ganguli et al. [19]	1996	O	O	O	O	O		O		
Inoue et al. [40]	2001	O	O	O						
Jensen et al. [51]	2006	O	O	O	O		O			
Katsumata et al. [44]	2007	O	O	O				O		
Choi et al. [28]	2012	O	O	O	O					
Cohen-Mansfield et al. [46]	2012	O	O	O	O	O			O	
Murayama et al. [29]	2012	O	O	O	O		O	O	O	
Takahashi et al. [31]	2015	O	O	O	O			O		
Umegaki et al. [23]	2015	O			O			O		
Koyama et al. [47]	2016	O	O	O	O				O	
Negron-Blanco et al. [30]	2016	O	O	O	O	O	O	O		
De-Rosende et al. [49]	2017	O	O	O	O				O	
Jing et al. [27]	2017	O	O	O	O		O	O		
Uemura et al. [2]	2018	O	O	O	O			O		
Xiang et al. [32]	2020	O	O	O	O	O	O	O	O	O

*Note.* HB = homebound; “O” means “yes”.

## Data Availability

The data that support the findings of this study are available from the corresponding author upon reasonable request.
